# Unexpected brain metastases from neuroendocrine prostate cancer detected by [^18^F]fluorocholine PET/CT: a case report

**DOI:** 10.3389/fmed.2026.1722967

**Published:** 2026-03-03

**Authors:** Yassir Benameur, Mohcine Hommadi, Omar Ait Sahel, Salah Nabih Oueriagli, Ikram Zahfir, Meryem Aboussabr, Jaafar El Bakkali, Abderrahim Doudouh

**Affiliations:** 1 Department of Nuclear Medicine, Mohammed V Military Teaching Hospital, Rabat, Morocco; 2 Faculty of Medicine and Pharmacy, Mohammed V University, Rabat, Morocco; 3 Department of Radiotherapy, Mohammed V Military Teaching Hospital, Rabat, Morocco

**Keywords:** [^18^F]fluorocholine PET/CT, brain metastases, case report, hybrid imaging, molecular imaging, neuroendocrine prostate cancer, small cell carcinoma

## Abstract

Neuroendocrine prostate cancer (NEPC) is a rare and aggressive variant of prostate carcinoma, often associated with atypical metastatic spread and poor prognosis. Brain metastases from NEPC are exceptional and may pose significant diagnostic challenges.

We report the case of a 59-year-old man referred for initial staging of prostate cancer after a transrectal ultrasound-guided biopsy that initially demonstrated poorly differentiated adenocarcinoma. Whole-body [^18^F]fluorocholine positron emission tomography/computed tomography (PET/CT) revealed subcentimetric hypermetabolic foci in the left frontal and left temporal cortex and in the left cerebellar hemisphere, suggestive of brain metastases. Pelvic lymphadenopathy was also identified, with no evidence of bone or other visceral involvement. Subsequent histopathological re-evaluation of the initial biopsy, including extended immunohistochemical analysis, demonstrated a neuroendocrine (small cell) component. The patient underwent stereotactic radiotherapy for the cerebral lesions and localized radiotherapy for the primary prostatic tumor.

This case underscores the diagnostic value of [^18^F]fluorocholine PET/CT in detecting unexpected metastatic sites in neuroendocrine prostate carcinoma. Early identification of central nervous system involvement may have therapeutic and prognostic implications, emphasizing the need for molecular imaging in atypical or aggressive prostate cancer phenotypes.

## Introduction

Prostate cancer typically metastasizes to the bones, regional lymph nodes, liver, and lungs. Brain metastases occur in less than 1% of cases (1). Neuroendocrine prostate cancer (NEPC), particularly its small cell carcinoma (SCC) subtype, represents a rare but highly aggressive histologic variant (2). Owing to its distinct biological behavior, SCC often presents with atypical clinical and biochemical features, including disproportionately low serum prostate-specific antigen (PSA) levels despite extensive disease burden, and a greater propensity for visceral and central nervous system metastases. These complicate diagnosis and therapeutic management (3).

## Case description

A 59-year-old man without significant prior medical history was referred to our department for initial staging of newly diagnosed prostate cancer. The diagnosis followed a transrectal ultrasound-guided biopsy performed after the patient presented with lower urinary tract symptoms. Twelve-core systematic sampling was obtained, and histopathological examination initially revealed a poorly differentiated adenocarcinoma (Gleason score 4 + 5 = 9), with immunohistochemical analysis pending at that time. The serum prostate-specific antigen (PSA) level was moderately elevated at 10.3 ng/ml.

**Figure 1:**
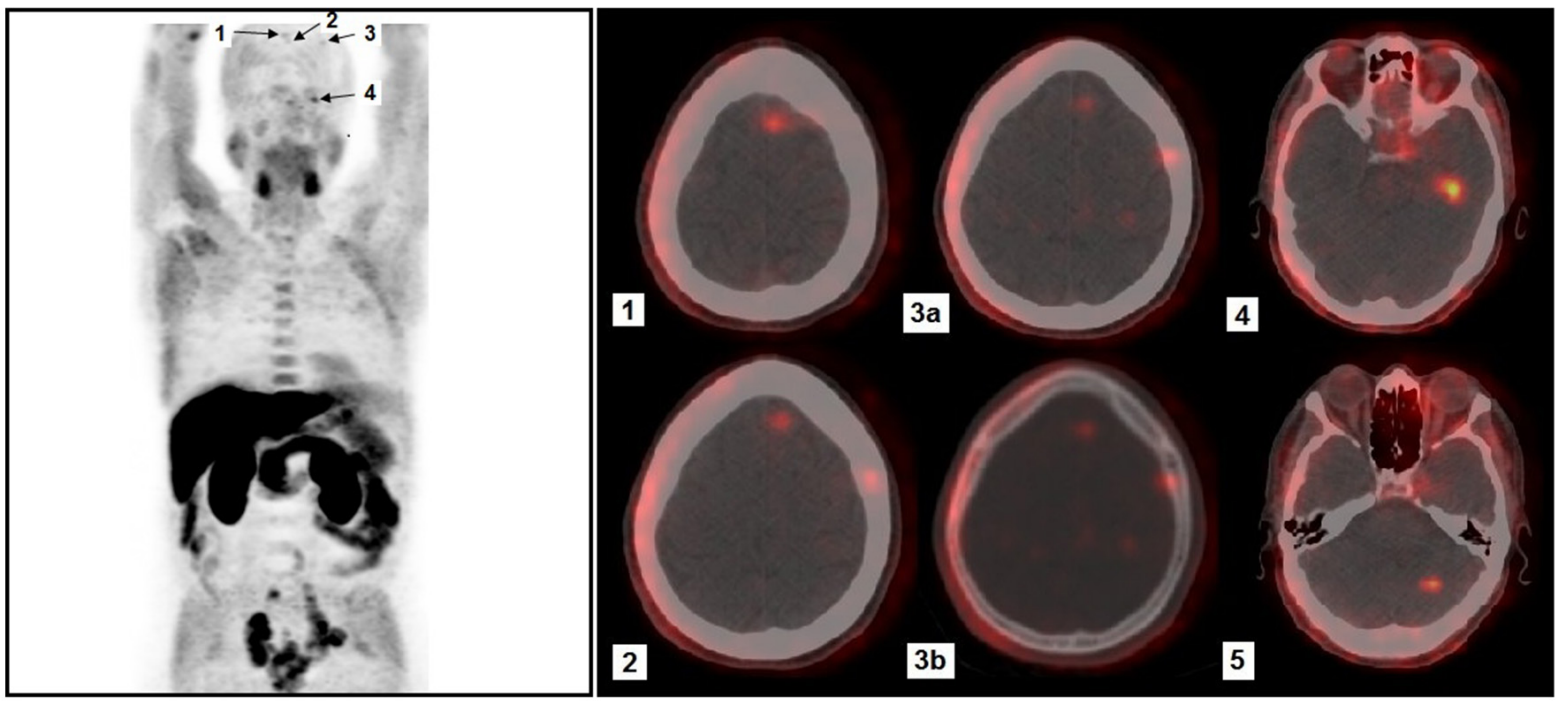
Whole-body maximum intensity projection (MIP) [18F]fluorocholine PET image **(left)** showing extensive pelvic lymph node involvement, as well as several intracranial foci (arrows). Physiologic tracer distribution is observed in the salivary glands, liver, pancreas and bowel. Axial fused PET/CT images **(right)** show intracranial foci: (**1, 2)** Two small paramedian foci located in the left frontal lobe at different axial levels, with very faint uptake. (**3)** Small focus located in the left lateral frontal lobe, with very low-level uptake; although it appears close to the skull on fused images, CT in bone window **(3b)** confirms the absence of skull involvement, indicating an intra-parenchymal lesion **(3a). (4)** Focus located in the left temporal lobe, demonstrating more conspicuous uptake. (**5)** Focus located in the left cerebellar hemisphere.

Whole-body [^18^F]fluorocholine positron emission tomography/computed tomography (PET/CT) was performed for staging. Imaging was acquired 60 min after the intravenous administration of 3.5 MBq/kg of [^18^F]fluorocholine, using a hybrid PET/CT system with low-dose CT for attenuation correction. The scan demonstrated increased tracer uptake in the prostate and pelvic lymph nodes. Notably, several subcentimetric hypermetabolic foci were observed in the left frontal lobe, left temporal lobe, and left cerebellar hemisphere, suggestive of cerebral metastases (Figure 1). A contrast-enhanced brain MRI was subsequently performed to further characterize these lesions. It showed small enhancing nodules in the corresponding locations, though some were less conspicuous than on PET/CT due to their small size. This highlighted the sensitivity of [^18^F]fluorocholine PET/CT for detecting small brain metastases in this clinical context. No osseous or other visceral lesions were identified on either PET/CT or CT of the chest, abdomen, and pelvis. Given the atypical metastatic pattern and the discordantly low PSA level relative to disease burden, the initial prostate biopsy was re-evaluated. Immunohistochemical staining revealed strong positivity for neuroendocrine markers, including synaptophysin and chromogranin A, with weak or absent PSA expression. Ki-67 was markedly elevated (>80%), further supporting a diagnosis of high-grade neuroendocrine carcinoma. These findings confirmed the presence of a primary neuroendocrine carcinoma component, consistent with small cell histology. The patient underwent stereotactic radiotherapy targeting the brain lesions and concurrent pelvic radiotherapy for local control of the primary tumor. Systemic platinum-based chemotherapy was given, in accordance with treatment protocols for extrapulmonary small cell carcinoma.

## Discussion

We describe a patient in whom [^18^F]fluorocholine PET was instrumental in uncovering brain metastases at the initial staging of a prostatic carcinoma, which turned out to have a small cell type neuroendocrine component. In the absence of histological confirmation of the brain metastases, alternative differential diagnoses to be considered included metastases from an occult extraprostatic primary tumor not visualized on [^18^F]fluorocholine PET, or primary brain tumors. However, the plurifocal nature of the lesions argued for metastatic lesions and the associated pelvic nodal disease suggested metastases from the prostatic tumor. Additional molecular imaging with somatostatin receptor PET could have provided complementary lesion characterization given the neuroendocrine phenotype, but was not obtained.

Brain metastases from prostate cancer are uncommon, with bones and lungs representing the pre-dominant sites of distant spread. The estimated incidence of intracranial involvement is less than 2%, and such metastases are more often identified post-mortem than during the patient's lifetime (4). When present, brain metastases typically occur in advanced stages of the disease, together with multiple other sites of metastasis. Only very rarely they represent an early manifestation, sometimes even preceding the diagnosis of the primary tumor. Small cell carcinoma (SCC) of the prostate, however, a rare and highly aggressive histologic subtype accounting for less than 1% of prostate malignancies, is more frequently associated with central nervous system (CNS) dissemination and carries a significantly poorer prognosis compared with conventional adenocarcinoma (5). SCC is characterized by rapid proliferation, early hematogenous dissemination, and a marked propensity for visceral metastases (2). It may arise *de novo*, as it did in the present case, or develop through neuroendocrine transdifferentiation of pre-existing adenocarcinoma, particularly under the selective pressure of prolonged androgen deprivation therapy (3). In many cases, SCC coexists with conventional adenocarcinoma components, forming histologically mixed tumors (3). The mixed and pure neuroendocrine phenotypes present significant diagnostic challenges, as they may be clinically suspected in aggressive disease yet remain difficult to confirm using conventional biomarkers and standard imaging alone. This phenomenon was evident in our patient, whose moderately elevated PSA level (10.3 ng/ml) was disproportionate to the extent of the disease present, including multiple pelvic lymph node metastases and brain metastases (6). In our case, the detection of cerebral lesions during initial staging prompted histopathological re-evaluation of the prostate biopsy, ultimately revealing small cell carcinoma. Our case highlights the importance of maintaining a high index of suspicion for neuroendocrine differentiation in patients with atypical metastatic patterns or discordant clinical and biochemical findings.

Although [^18^F]fluorocholine PET/CT is primarily employed to assess local recurrence and bone metastases in prostate cancer (7), uptake of [^18^F]fluorocholine in cerebral or cerebellar metastases has previously been described (8, 9). In contrast to the patient we report here, both of these cases were longstanding prostatic carcinomas (3 and 11 years, respectively). In the case of Gizewska (8), multiple metastases in lungs, chest lymph nodes, bone and subcutaneous lymph nodes were present at the time brain metastases were documented, while the cerebellar metastasis published by Imperiale (9) was the only metastasis found. In the latter case, this metastasis was histologically confirmed to be from prostatic adenocarcinoma, but the prostatic carcinoma had been shown to be poorly differentiated at first diagnosis (Gleason 9), similar to our case. Our case shows that [^18^F]fluorocholine PET is able to depict metastases from neuroendocrine prostatic carcinoma as well.

In our patient, the detection of cerebral metastases during routine staging provided information that guided further diagnostic and therapeutic decision-making. The prognosis of patients with SCC of the prostate and brain metastases remains poor, with median survival often reported to be less than 6 months despite aggressive treatment combining chemotherapy, radiotherapy, or stereotactic radiotherapy (10). Early identification of neuroendocrine differentiation profoundly influences management. While androgen deprivation therapy remains the cornerstone of conventional prostate cancer treatment, SCC is typically refractory to hormonal therapy and requires systemic chemotherapy, most commonly platinum-based regimens modeled after small cell lung cancer protocols (11).

## Conclusion

This case illustrates the rare but clinically significant occurrence of brain metastases in neuroendocrine prostate cancer. It underscores the importance of histopathological reassessment in patients with atypical imaging or biochemical profiles and emphasizes the diagnostic value of [^18^F]fluorocholine PET/CT in identifying unexpected metastatic sites. A multidisciplinary approach integrating advanced imaging, pathology, and individualized treatment planning is essential to optimize management in such aggressive and uncommon disease presentations.

## Data Availability

The original contributions presented in the study are included in the article/supplementary material, further inquiries can be directed to the corresponding author.
